# Increased cerebral blood flow in the right frontal lobe area during sleep precedes self-awakening in humans

**DOI:** 10.1186/1471-2202-13-153

**Published:** 2012-12-21

**Authors:** Sayaka Aritake, Shigekazu Higuchi, Hiroyuki Suzuki, Kenichi Kuriyama, Minori Enomoto, Takahiro Soshi, Shingo Kitamura, Akiko Hida, Kazuo Mishima

**Affiliations:** 1 Department of Psychophysiology, National Institute of Mental Health, National Center of Neurology and Psychiatry, Tokyo, 187-8502, Japan; 2 Japan Society for the Promotion of Science, Tokyo, 102-8471, Japan; 3 Department of Somnology, Tokyo Medical University, Tokyo, 160-0023, Japan; 4 Department of Life Sciences and Bio-informatics, Graduate School of Allied Health Sciences, Tokyo Medical and Dental University, Tokyo, 113-0034, Japan; 5 Department of Human Science, Faculty of Design, Kyushu University, Fukuoka, 815-8540, Japan; 6 Department of Adult Mental Health, National Institute of Mental Health, National Center of Neurology and Psychiatry, Tokyo, 187-8502, Japan

**Keywords:** Time estimation ability, Self-awakening, Sleep, Cognitive science, Prefrontal cortex, Insomnia

## Abstract

**Background:**

Some people can subconsciously wake up naturally (self-awakening) at a desired/planned time without external time stimuli. However, the underlying mechanism regulating this ability remains to be elucidated. This study sought to examine the relationship between hemodynamic changes in oxyhemoglobin (oxy-Hb) level in the prefrontal cortex and sleep structures during sleep in subjects instructed to self-awaken.

**Results:**

Fifteen healthy right-handed male volunteers with regular sleep habits participated in a consecutive two-night crossover study. The subjects were instructed to wake up at a specified time (“request” condition) or instructed to sleep until the morning but forced to wake up at 03:00 without prior notice (“surprise” condition). Those who awoke within ± 30 min of the planned waking time were defined as those who succeeded in self-awakening (“success” group). Seven subjects succeeded in self-awakening and eight failed.

No significant differences were observed in the amounts of sleep in each stage between conditions or between groups. On the “request” night, an increase in oxy-Hb level in the right prefrontal cortex and a decrease in δ power were observed in the “success” group around 30 min before self-awakening, whereas no such changes were observed in the “failure” group. On the “surprise” night, no significant changes were observed in oxy-Hb level or δ power in either group.

**Conclusions:**

These findings demonstrate a correlation between self-awakening and a pre-awakening increase in hemodynamic activation in the right prefrontal cortex, suggesting the structure’s contribution to time estimation ability.

## Background

We are often able to wake up spontaneously at a desired or planned time or to subconsciously estimate time elapsed during sleep without receiving external time stimuli such as from an alarm clock. This phenomenon is referred to as self-awakening [1-5] or anticipated sleep termination [6], and it is believed to be achieved by time estimation ability (TEA) [7], which is exercised even during sleep [8,9]. A previous study reported that more than half of healthy adults believe that they are able to wake up by themselves during habitual sleep with an accuracy (i.e., difference between planned and actual wake up time) of ± 10 min [10].

Previous studies have identified several factors related to TEA during sleep, including an increased adrenocorticotropic hormone (ACTH) level observed before awakening [6], recurrent semi-awakening during sleep [11,12], differences in preceding sleep structures [13,14], diurnal fluctuation [15,16], and psychological motivation [3,17]. However, the brain region responsible for TEA has not been identified. Meanwhile, considerable information has been obtained regarding the neurological basis of TEA while awake [18-21]. The basal nuclei (corpus striatum), cerebellum, and prefrontal cortex have been shown to contribute to TEA in the order of seconds to minutes, and each of these three regions plays a different role. The cerebellum processes mainly timing information [22-24], the basal nuclei, in particular the corpus striatum, functions as a core timer [25-27], and the prefrontal cortex assists the basal nuclei in controlling timer rate, attention allocation, and temporal memory processing [26,28-30]. Despite the possible contributions of these brain regions and the interval timer system to TEA during sleep, no study has continuously monitored the activity of these regions during the course of natural sleep. Thus, the neurological basis of TEA during sleep remains to be elucidated.

Several recent studies have shown changes in cerebral hemodynamics during sleep with near infrared spectroscopy (NIRS) [31-35]. One study showed a decrease in oxy-Hb levels of 5–10% from baseline (before sleep onset) during the first 60 min of sleep [32,33]. This technique has the potential to elucidate specific hemodynamic change during self-awakening. The objective of this study was to determine whether the prefrontal cortex mediates self-awakening by: 1) setting two night conditions (“request”, where subjects were instructed at bedtime to wake up 3 h after lights out, earlier than the regular wake time; and “surprise”, where subjects were instructed to sleep for 8 h after lights out but forced to wake up 3 h after lights out without prior notice); and 2) measuring hemodynamic changes to the prefrontal cortex over time by multi-channel NIRS while simultaneously measuring sleep structures by polysomnography (PSG) during the first 3 h after lights out.

## Methods

### Participants

Fifteen healthy, right-handed male volunteers (age 22.1 ± 0.7 years (mean ± SD)) with regular sleep habits participated in the consecutive two-night, single-blind, crossover conditions (“request” and “surprise”). All subjects provided written informed consent after being informed of the possible risks and details of the study. A physician and a psychiatrist examined the subjects and found that no subjects suffered from a neurological or psychiatric disorder or had a history of psychoactive drug use. Subjects were instructed to maintain a regular sleep-wake schedule, record their sleep patterns in a sleep log, and abstain from caffeine, nicotine, and alcohol for 1 week prior to the experiment. All subjects wore a wrist activity recorder (Actiwatch-L, Mini-Mitter Co., Inc. Bend, OR) for 1 week prior to the experiment. Sleep onset and offset times were determined using Actiware Sleep software (V3. 2 Mini-Mitter Co., Inc.). Data recorded in the sleep logs together with sleep onset and offset times were used to confirm regular sleep-wake schedules. The study protocol was approved by the Ethics Committee of the Institutional Review Board of the National Center of Neurology and Psychiatry, Japan.

### Experimental procedures

**Figure 1:**
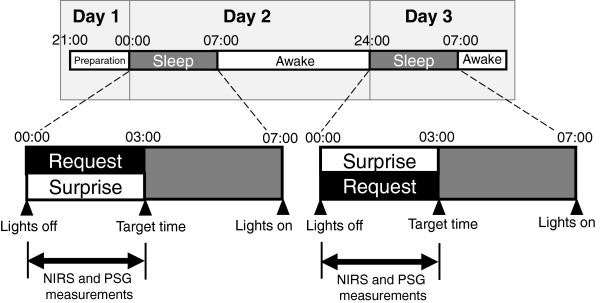
**Experimental schedule.** Subjects arrived at the sleep laboratory around 21:00 and were instructed to go to bed at 00:00 (lights off) after preperations were made to measure PSG and NIRS. Experiments under each of the two conditions were conducted twice for each subject in a consecutive two-night, single-blind crossover setting (“request” and “surprise”) without notice of which condition would be examined first. Each subject was randomly allocated to one of two conditions and instructed at bedtime to wake up at: 1) 03:00 (“request”); or 2) 08:00, but was unexpectedly woken at 03:00 (“surprise”). When the subject could self-awaken at 03:00 ± 30 min, the result was recorded as “success” or otherwise as “failure”. Each subject was examined under the other condition the following day.

All experiments were performed in the time isolation laboratory at the National Center of Neurology and Psychiatry in Japan. Room temperature and humidity were controlled at 24°C and 60%, respectively. Subjects arrived at the sleep laboratory at 21:00 and were instructed to go to bed at 00:00 (Lights off) after electrodes and sensors for PSG and NIRS were attached. Each subject was randomly allocated to the “request” or “surprise” sessions on a within-subject crossover basis as follows: 1) “request”: subjects were instructed at bedtime to wake up at 03:00; and 2) “surprise”: subjects were instructed at bedtime to wake up at 08:00, but were unexpectedly woken at 03:00 (Figure 1). When a subject could self-awaken at 03:00 ± 30 min, the event was recorded as “success”. When a subject could not awaken spontaneously at 03:00 ± 30 min, the event was recorded as “failure”. Each subject was examined under the other condition the following night.

### Measurement

Simultaneous measurement of PSG and NIRS was performed throughout the sleep period, which was defined as the period from sleep onset to the designated wake time defined for each condition. PSG comprised electroencephalography (EEG; Fp1-A2, Fp2-A1, F3-A2, F4-A2, C3-A2, C4-A1, O1-A2, and O2-A1) in accordance with the 10–20 electrode system, electrooculography (EOG; left-A2 and right-A1), chin surface electromyography (chin-EMG), and electrocardiography (ECG). PSG data were obtained continuously during each experiment, digitalized at 500 Hz, and stored in a digital EEG system (Comet, Grass Technologies, Astro-Med, Inc., RI). The high cut and low cut filters were set at 60 Hz (except chin-EMG at 120 Hz) and 0.5 Hz, respectively.

NIRS of the human brain is a relatively flexible technique for measuring hemodynamic changes on the basis of differences in the absorbance of harmless near-infrared light between oxyhemoglobin (oxy-Hb) and deoxyhemoglobin (deoxy-Hb), and NIRS has been used to elucidate the physiological mechanisms of the blood oxygen level-dependent response [36-42]. Previous studies showed that NIRS signals correlate strongly with functional magnetic resonance imaging (fMRI) measurements [42,43].

In our study, regional hemodynamic changes in brain tissue were monitored using a continuous wave-type fNIRS system (OMM-3000, Shimadzu Corporation, Kyoto, Japan), which consisted of three semiconductor laser diodes producing wavelengths of 780, 805, and 830 nm. This system is used to calculate changes in oxy-Hb, deoxy-Hb, and total-Hb levels on the basis of the modified Beer-Lambert equation as a function of light absorbance of Hb and pathlength [44,45]. Since the used wavelengths are in a relatively narrow range, their corresponding pathlengths are assumed to be approximate to those of the algorithm used in the OM-3000 system. Thus, changes in oxy-Hb levels were calculated using changes in absorption (Δabs) and the absorption coefficient of each wavelength, but without the differential pathlength factor (DPF) for adjustment [46,47]. The relevant equations are as follows:

(1) $$\begin{array}{c} \Delta \mathrm{oxy}-\mathrm{Hb}=\left(-1.4887\right)\times \Delta \mathrm{abs}\left[780\;\mathrm{nm}\right]\\ \;+0.5970\times \Delta \mathrm{abs}\left[805\;\mathrm{nm}\right]\\ \;+1.4847\times \Delta \mathrm{abs}\left[830\;\mathrm{nm}\right] \end{array}$$

(2) $$\begin{array}{c} \Delta \mathrm{deoxy}-\mathrm{Hb}=1.8545\times \Delta \mathrm{abs}\left[780\;\mathrm{nm}\right]\\ \;+\left(-0.2394\right)\times \Delta \mathrm{abs}\left[805\;\mathrm{nm}\right]\\ \;+\left(-1.0947\right)\times \Delta \mathrm{abs}\left[830\;\mathrm{nm}\right] \end{array}$$

(3) Δtotal−Hb=Δoxy−Hb+Δdeoxy−Hb

The coefficients in equations (1), (2), and (3) are coefficients of absorption at corresponding wavelengths. Determining changes in Hb levels with the modified Beer-Lambert Law depends on DPF, but optical pathlengths differ among individuals and depend on the position within the brain. Thus, oxy-Hb values measured by the OMM-3000 system show relative change in concentration, as opposed to absolute Hb concentration.

**Figure 2:**
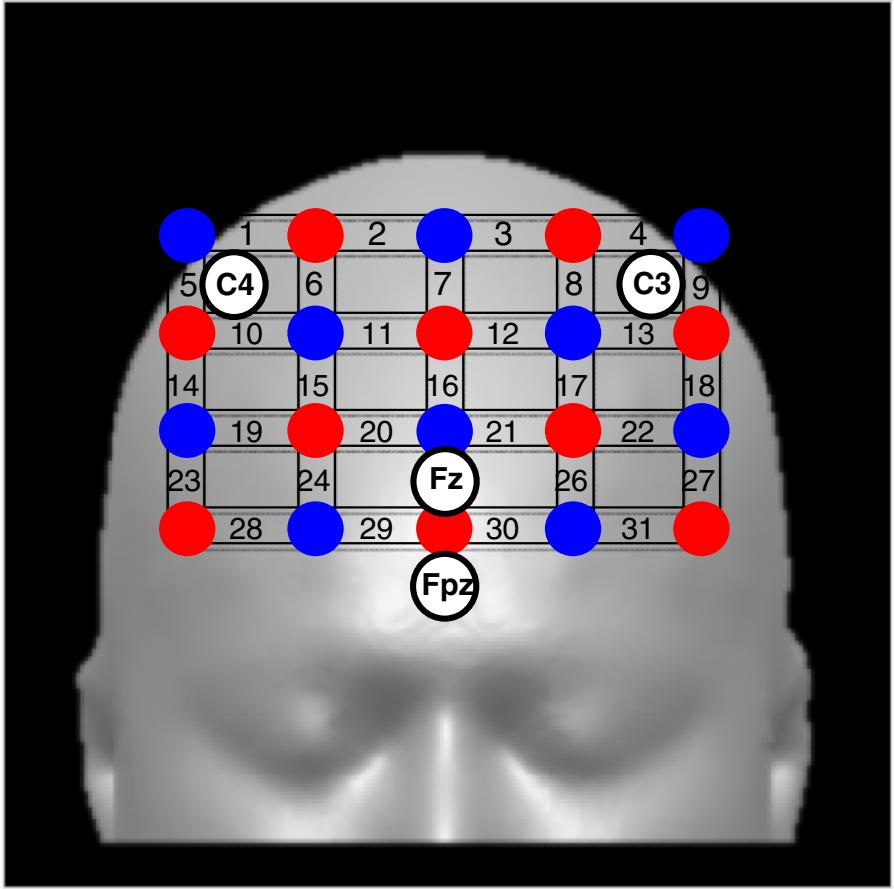
**Approximate positions of the 31 fNIRS channels superimposed on a head model.** The 31 measuring channels were produced by optrodes placed equidistantly over the prefrontal cortex (PFC) area. The lower line of the 4 × 5 optrode probe was positioned along the reference curve linking C3, C4, Fz, and FPz. Red denotes source optrodes and blue denotes detector optrodes.

Optrodes for producing near-infrared light (n=10) and for collecting transmitted light (n=10) were placed alternately at 3-cm intervals in a 4 × 5-cm square on the frontal cortex (FC) area (Fpz, Fz, C3, and C4 were used to adjust the position of the optrodes), resulting in 31 channels for measuring each Hb level every 200 ms (Figure 2). The energy emitted by each laser diode during long-term exposure was well below the United States safety level (Class 1 M) [48]. Subject behavioral status and sleep-wake status, as well as hemodynamic status, were continuously and carefully monitored visually by two well-trained research attendants. In our study subjects, we confirmed the following: 1) oxy-Hb levels were stable for at least 10 min before lights-off on the experimental night in a recumbent position on the bed; and 2) subjects showed decreased oxy-Hb levels of 5–10% from baseline (before sleep onset) during the first 60 min of sleep, which is consistent with findings in previous studies [32,33].

### Data analysis

NIRS data could not be obtained from 4 of 15 subjects due to displacement of light-producing optrodes during sleep. Evaluation of sleep structures was made for 15 subjects while analysis of sleep structures in relation to oxy-Hb was performed for the 11 subjects with available NIRS data.

PSG data obtained over a period of about 3 h were scored for 30-s epoch periods according to standard criteria [49]. The amount of stage W, stage 1, stage 2, stage 3+4, and stage REM sleep, as well as sleep latency, wake after sleep onset (WASO), and arousal index [35,50] for total sleep time were calculated for all PSG recordings. Slow-wave sleep was calculated as the sum of stage 3 and stage 4 sleep. The frequency of body movement during sleep was determined by viewing a monitor and EEG waveforms in real-time. For more detailed analysis of sleep structures, power spectral analysis using fast Fourier transform (FFT) as a parameter of brain activity at the cortical level was also examined with Sleep Sign (Kissei Comtec, Nagano, Japan). The EEG power (delta: 0.5–4 Hz; theta: 4–8 Hz; alpha: 8–13 Hz, and sigma: 13–15 Hz) of F3-A2 and F4-A1 was obtained by FFT analysis of 2048 data-points (8 × 4.096-s sequences) tapered by a Hanning window. Since the eight sequences are longer than the visual epoch definitions for sleep staging, both sides of each sequence overlapped by 0.39 s. FFT values for each sequence were summed to obtain the value of EEG power per epoch. Epochs containing artifacts were visually identified and excluded from the analysis.

We focused on changes of oxy-Hb in the 60- to 30-min period before self-awakening, since previous study demonstrated increases in adrenocorticotropic hormone levels during this period [6]. NIRS analysis on the “request” night was performed using data obtained from each subject during the final 30 min before self-awakening. On the “surprise” night, data obtained during the same period as that on the “request” night were used for analysis. NIRS data obtained from multiple analyzable channels every 200 ms were averaged every 30 s in line with PSG analysis to calculate Hb levels after excluding visible artifacts. A 0.02–1 Hz band pass filter was applied to individual data to remove artifacts caused by high-frequency instrument noise and low-frequency drift in oxy-Hb and deoxy-Hb signals in the fNIRS system. Since oxy-Hb level determined by NIRS is the most sensitive and representative optimal indicator of regional hemodynamic changes, we used this parameter in our analysis [32,36,51]. For the analysis of each right and left FC (rPFC and lPFC) region, we averaged the channels separately for each side (right and left) and region (rPFC region: 14, 15, 19, 20, 23, 24, 28, and 29; and lPFC region: 17, 18, 21, 22, 26, 27, 30, and 31).

### Statistical analysis

To determine the effect of the order of each experimental condition on sleep duration during each PSG period, we compared the nocturnal duration of each sleep stage between the groups under each experimental condition using two-way measures ANOVA. We also analyzed the rate of oxy-Hb changes between the groups under the each experimental condition using two-way ANOVA (experimental condition × group). To compare fluctuations in oxy-Hb levels, individual sleep structures, and EEG power with time between the groups under each experimental condition, three-way (1 between-subjects factor and 2 within-subjects factors) repeated measures ANOVA (experimental condition × results group × time course) was performed. If significant interactions were obtained, planned follow-up ANOVA and post-hoc analysis were subsequently conducted to verify our assumption that changes in hemodynamics and EEG power in the prefrontal cortex area mediates self-awakening. SPSS ver. 18.0 (IBM, Cary, NC) was used for all statistical analysis. Results are expressed as mean and standard error values. Significance was set at *p*<0.05.

## Results

### Self-awakening success and failure

In this study, 7 of 15 subjects succeeded in waking up at the predetermined time (03:00) under the “request” condition.

### Sleep structures

Table 1 shows sleep parameters for the full sleep period (3 h) after lights-out until self-awakening or forced awakening and the final 30 min before waking in the 15 subjects by condition (“request” vs. “surprise”) and group (“success” vs. “failure”). Two-way ANOVA (experimental condition × group) revealed no significant differences in any of these parameters for any of the sleep periods or between the groups or conditions.

**Table 1:** Sleep structures over 3 h and final 30 min

		**Success (n=7)**		**Failed (n=8)**		**Condition**	**Results**
		**Request**	**Surprise**	**Request**	**Surprise**	**F [1, 13] (p-value)**	**F [1, 13] (p-value)**
3 h	Wake (min)	14.14 ± 13.70	19.79 ± 11.10	38.31 ± 12.82	26.00 ± 10.38	0.15 (0.70)	1.05 (0.32)
	aS1 (min)	7.86 ± 2.62	6.29 ± 3.83	11.81 ± 2.45	16.94 ± 3.58	0.41 (0.53)	4.25 (0.06)
	bS2 (min)	52.86 ± 10.69	43.14 ± 7.53	55.56 ± 10.00	69.06 ± 7.05	0.17 (0.69)	1.49 (0.25)
	cSWS (min)	71.00 ± 12.67	58.57 ± 11.88	51.38 ± 11.85	50.63 ± 11.12	1.49 (0.24)	0.75 (0.40)
	dREM (min)	15.79 ± 5.19	14.00 ± 5.23	22.31 ± 4.85	9.13 ± 4.89	3.12 (0.10)	0.02 (0.89)
	eTST (min)	147.50 ± 17.29	122.00 ± 12.83	141.06 ± 16.17	145.75 ± 12.01	1.01 (0.33)	0.23 (0.64)
	SleepLatency (min)	6.14 ± 2.27	3.29 ± 1.19	4.50 ± 2.12	4.00 ± 1.11	1.25 (0.77)	0.06 (0.82)
	fWASO (min)	14.29 ± 15.14	16.86 ± 11.69	36.38 ± 14.16	24.25 ± 10.94	0.31 (0.28)	0.80 (0.39)
	Arousal Index (h)	7.09 ± 1.98	9.01 ± 2.14	6.61 ± 1.83	7.71 ± 1.98	1.32 (0.27)	0.13 (0.73)
	Number of Movements	6.33 ± 2.36	6.17 ± 2.08	7.86 ± 2.19	5.71 ± 1.92	1.08 (0.32)	0.04 (0.85)
Final 30 min							
	Wake (min)	3.07 ± 1.09	4.43 ± 1.98	4.13 ± 2.04	8.88 ± 3.09	1.19 (0.29)	0.39 (0.54)
	aS1 (min)	1.57 ± 0.95	0.93 ± 0.37	0.56 ± 0.29	2.50 ± 1.60	0.54 (0.47)	0.06 (0.80)
	bS2 (min)	5.14 ± 2.06	12.79 ± 3.52	7.69 ± 2.88	11.31 ± 3.99	3.38 (0.09)	0.02 (0.88)
	cSWS (min)	9.93 ± 4.25	9.00 ± 2.84	7.38 ± 4.07	3.19 ± 2.45	0.95 (0.35)	1.01 (0.33)
	dREM (min)	3.71 ± 2.52	0.50 ± 0.36	7.88 ± 3.81	4.13 ± 3.59	1.04 (0.33)	2.17 (0.16)
	eTST (min)	20.36 ± 4.15	23.21 ± 4.13	23.50 ± 4.15	21.13 ± 4.60	0.02 (0.89)	0.01 (0.93)

Request: experimental condition where subjects were instructed at bedtime to wake up at 03:00.

Surprise: experimental condition where subjects were instructed at bedtime to wake up at 08:00, but were unexpectedly woken at 03:00.

Success: when a subject could self-awaken at 03:00 ± 30 min.

Failure: when a subject could not awaken spontaneously at 03:00 ± 30 min.

^a^stage 1 sleep; ^b^S2: stage 2 sleep; cSWS: stage 3+4 sleep; ^d^REM: stage rapid eye movement sleep; ^e^TST: total sleep time; ^f^WASO: wake after sleep onset. *p < 0.05.

### Thirty minutes before waking

#### Temporal changes in oxy−Hb levels in the PFC region

**Figure 3:**
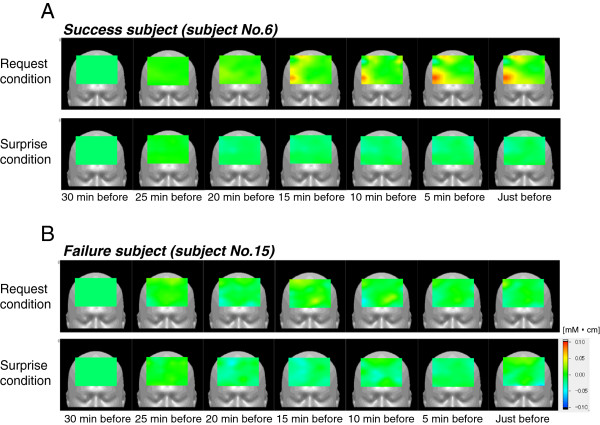
**Change of oxy-Hb level during 30 min before waking in one “success” and one “failure” subject.** Upper panel shows the gradual increase in oxy-Hb level in the right prefrontal region of a “success” group subject on the “request” night but not on the “surprise” night (**A**). In contrast, the oxy-Hb level showed no increase under either condition in the “failure” subject (**B**).

Figure 3 shows the change in oxy-Hb level during the 30-min period before waking in one “success” and one “failure” subject. The upper panel shows that the oxy-Hb level in the rPFC area of the “success” subject gradually increased under the “request” condition, but not under the “surprise” condition (Figure 3A). In contrast, in the “failure” subject, the oxy-Hb level showed no increase under either condition (Figure 3B).

**Figure 4:**
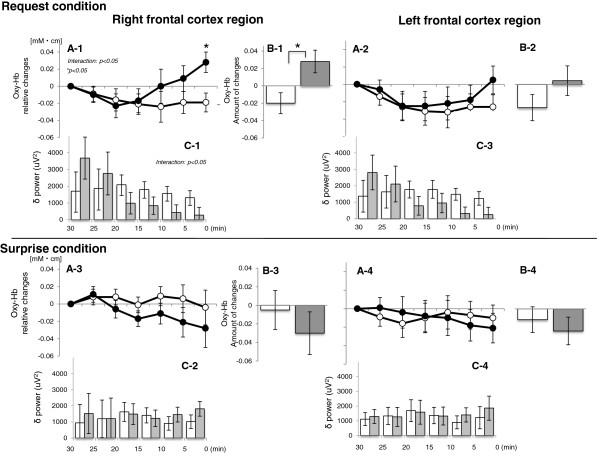
**Change of oxy-Hb level and sleep structures during the final 30 min before waking (n=11).** Temporal change in the oxy-Hb level (**A**), the rate of oxy-Hb levels (**B**), and temporal change δ power in the prefrontal cortex (PFC) region (**C**) during the final 30 min before waking in 11 subjects. The “request” condition and oxy-Hb level (change according to time course and the rate during the final 30 min before waking) in the right PFC (rPFC) region gradually increased 20 min before predominant waking in the “success” group (*black circle*) compared with the “failure” group (○) (**A**-**1**, **B**-**1**). The delta power of the “success” group also decreased in the rPFC region with time under the request condition (**C**-**1**).

Figure 4A shows changes in oxy-Hb levels in the rPFC and lPFC area of 11 subjects during the final 30 min of sleep under each condition. In the rPFC area, three-way ANOVA (condition × time course × group) showed a significant second-order interaction (condition × time course × group: *F*_(6,54)_ = 2.813, p = 0.019). Planned follow-up two-way repeated ANOVA (time course × group) revealed that the oxy-Hb level under the “request” condition increased gradually 20 min before predominant waking in the “success” group compared with the “failure” group, and a significant interaction of time course × group was observed (*F*_(6,54)_ = 3.345, p = 0.007). Additional follow-up one-way ANOVA revealed that oxy-Hb level increased significantly with time in the “success” group (*F*_(5,54)_ = 7.238, *p* = 0.016), but not in the “failure” group (*F*_(5,54_ = 2.154, *p* = 0.113). The post-hoc test confirmed a significant difference between groups at 5 min before predominant waking (Bonferroni, p = 0.001) (Figure 4A-1). Under the “surprise” condition, follow-up two-way ANOVA revealed that fluctuation of oxy-Hb levels did not differ with time between the “success” and “failure” groups (interaction: *F*_(1,9)_ = 0.801, *p* = 0.574; time course: *F*_(6,54)_ = 1.675, *p* = 0.145; group: *F*_(1,9)_ = 1.200, *p* = 0.302) (Figure 4A-3). In the lPFC area, oxy-Hb level did not show any significant differences (condition: *F*_(1,9)_ = 1.226, *p* = 0.297; time course: *F*_(6,54)_ = 2.225, *p* = 0.098; group: *F*_(1,9)_ = 0.102, *p* = 0.757) nor an interactions (*F*_(6,54)_ = 1.556, *p* = 0.178) (Figure 4A-2, A-4).

For oxy-Hb changes over 30 min in the rPFC area, a significant interaction of condition × group was obtained (*F*_(1,9)_ = 3.513, *p* = 0.034) (Figure 4B-1, B-3). Under the “request” condition, the additional post-hoc test revealed that the rate of oxy-Hb change in the “success” group increased significantly more than in the “failure” group (*F*_(1,9)_ = 7.768, *p* = 0.021), but not under the “surprise” condition (*F*_(1,9)_ = 0.668, *p* = 0.435). Meanwhile, in the lPFC area, no difference in oxy-Hb change was observed (condition: *F*_(1,9)_ = 0.275, *p* = 0.021; group: *F*_(1,9)_ = 0.287, *p* = 0.605; interaction: *F*_(1,9)_ = 2.183, *p* = 0.174) (Figure 4B-2, B-4).

#### Temporal changes in sleep structures

Two-way ANOVA revealed a significant interaction of time course × group under the “request” condition (interaction: *F*_(5,45)_ = 3.037, *p* = 0.019; time course: *F*_(5,45)_ = 0.494, *p* = 0.779; group: *F*_(1,9)_ = 1.833, *p* = 0.209), but not under the “surprise” condition (interaction: *F*_(5,45)_ = 1.134, *p* = 0.356; time course: *F*_(5,45)_ = 0.867, *p* = 0.511; group: *F*_(1,9)_ = 0.902, *p* = 0.367). Moreover, additional one-way ANOVA confirmed a significant increase in REM stage sleep in the “success” group with time under the “request” condition (time course: *F*_(5,45)_ = 2.739, *p* = 0.043). Fluctuations of values in other sleep stages did not differ between condition or group.

#### Temporal changes in δ power

Figure 4-C shows changes in δ power in the lPFC and rPFC area during the final 30 min of sleep under each condition. In the rPFC region, three-way ANOVA showed a significant interaction of time course × group (*F*_(5,45)_=3.426, *p*=0.010). Follow-up one-way ANOVA revealed that δ power decreased significantly with time in the “success” group (*F*_(5,45)_ = 3.113, *p* = 0.031), but not in the “failure” group (*F*_(5,45)_ = 0.485, *p* = 0.784) (Figure 4C-1, C-3). In the lPFC region, three-way ANOVA showed no significant difference with time between conditions and groups (condition: *F*_(1,45)_ = 0.001, *p* = 0.972; time course: *F*_(1,45)_ = 1.739, *p* = 0.145; group: *F*_(5,45)_ = 0.014, *p* = 0.908) or interaction (p ≥ 0.05 for all) (Figure 4C-2, C–4).

## Discussion

We examined changes in oxy-Hb levels during sleep as well as sleep depth and amount over time until a planned wake time under the “request” and “surprise” conditions by simultaneously recording NIRS and PSG data. The results revealed that under the “request” condition, an increase in the oxy-Hb level in the right prefrontal region and a rapid decrease in δ power occurred during the final 30 min of sleep in subjects who succeeded in self-awakening, whereas no changes were observed in these three parameters in subjects who failed to self-awaken. Under the “surprise” night condition, no significant changes were observed in these parameters during the final 30 min of sleep in either group.

### Success rate for self-awakening

Seven of the 15 subjects succeeded in self-awakening. A previous telephone survey involving 300 subjects showed that more than half of the subjects were able to wake up naturally without an alarm clock [3]. Hall and Brush tested whether subjects could wake up at a predetermined time on 50–100 nights and reported that about 70% were able to wake up almost exactly at the planned time [52,53]. The self-awakening success rate in the present study is almost identical to that in previous studies.

### Sleep structures preceding self-awakening

Although the neurological mechanism mediating self-awakening has not been clarified, it has been postulated that strong motivation toward self-awakening and resulting psychological strain reduces slow-wave sleep and increases shallow sleep, resulting in a lowered arousal threshold [54]. Previous studies of sleep structures preceding self-awakening have yielded discrepant results; some showed a decrease in sleep depth [1,54], while others showed no change [6]. In the present study, assessment of sleep structures over the entire period between lights out and self-awakening showed no significant difference between the “request” and “surprise” nights. Meanwhile, the analysis results of sleep structures immediately before self-awakening and those of power analysis had several important implications. First, it should be noted that subjects who succeeded in self-awakening showed an increase in REM sleep over time during the final 30 min of sleep on the “request” night. However, the total amount of REM sleep during the period did not differ between conditions, and no such increase was observed in subjects who failed. Since REM sleep tends to occur at around 3 h after lights out—in other words, at the planned wake time—it is not surprising that REM sleep occurred during the final 30 min before self-awakening. Nevertheless, the fact that increased REM sleep was observed just before self-awakening in the “success” group under the “request” condition, but not under the “surprise” condition, supports the contention that increased REM sleep facilitates successful self-awakening [10,13]. Second, it should be noted that a rapid decrease in δ power was also observed in the same area during the final 30 min of sleep in the “success” group under the “request” condition. This suggests that the rapid decrease in δ power around the planned time of self-awakening and the associated reciprocal increase in REM sleep may contribute to successful self-awakening.

### Mechanism and localization of self-awakening

Subjects who succeeded in self-awakening showed an increase in oxy-Hb level in the right prefrontal region before self-awakening on the “request” night, which appears to reflect increased blood flow in the region. However, no significant differences were seen between groups for frequency of body movement, duration of each sleep stage, WASO, or total sleep time during the same period. It is thus unlikely that the observed increase in oxy-Hb level before self-awakening was caused by a change in the oxy-Hb level in association with coarse fluctuation of sleep structures or body movement. Subjects who succeeded in self-awakening showed decreased δ power and increased REM sleep during the final 30 min of sleep on the “request” night. It is likely that these changes in sleep structures secondarily or simultaneously led to a change in local blood flow in the prefrontal region [55]. It is therefore reasonable to assume that increased blood flow in the prefrontal region resulted in self-awakening and the consequent arousal reaction. However, it remains unclear in our study why the blood flow increased significantly in the right but not the left prefrontal region.

It has been suggested that self-awakening is achieved by some kind of drive related to time recognition during sleep [8]. The present findings support this contention indirectly. At least in the awake state, the basal nuclei (corpus striatum), cerebellum, and right prefrontal region have been shown to contribute to TEA [24,26,27]. The right prefrontal region, in which increased oxy-Hb levels were observed, is a region that showed increased blood flow in awake subjects during a time recognition test [30,56], suggesting the contribution of increased blood flow in this region to successful self-awakening. However, the kind of physiological factors that trigger the increase in REM sleep or blood flow in the right prefrontal region during the 30 min before awakening in the present study remain unclear. Born et al. observed an increased ACTH level about 1 h prior to anticipated sleep termination [6]. Positron emission tomography (PET) studies that have examined the correlation between cerebral blood flow, ACTH, and cortisol levels while awake have demonstrated that increased secretion of ACTH and cortisol correlates with increased blood flow [57-60]. Subjects in the present study who succeeded in self-awakening might also have had increased the activity of the hypothalamic-pituitary-adrenal axis before self-awakening.

The present findings suggest the involvement of pre-awake activation of the right prefrontal region in self-awakening. The effect of heart rate and peripheral vasodilation on cerebral blood flow is important when interpreting the present data. Such factors are expected to have various effects on the measured responses under different conditions (“request” versus “surprise”) and in different groups (“success” versus “failure”). Therefore, it is likely that heart rate and peripheral vasodilation played specific roles in the “success” group under the “request” condition. In addition, the sample population was relatively small, due to highly complex procedures involving spontaneous PSG and NIRS over the period of 3 h during sleep. Findings of this study need to be confirmed by other methods such as PET and fMRI. Once confirmed, further studies using techniques such as simultaneous measurement of fMRI and sleep EEG are needed to determine whether the deep brain regions, which are considered to be related to TEA and include the basal nuclei (striatum) and cerebellum, are involved in achieving self-awakening. Moreover, it will be necessary to elucidate whether TEA in the order of hours during sleep occurs in the same manner as TEA in the order of seconds to minutes. From the clinical viewpoint, patients who repeatedly experience sleep state misperception/paradoxical insomnia [61-63], waking after sleep onset, or waking in the early morning may have excessive and persistent activation of the right prefrontal region, which is caused by undefined mechanisms during sleep. Thus, elucidation of the neural mechanism of self-awakening should contribute to elucidating the pathophysiology of such difficult-to-treat insomnia symptoms.

## Conclusions

Our present findings demonstrate a correlation between self-awakening and a pre-awakening increase in hemodynamic activation in the right prefrontal cortex, suggesting the structure’s contribution to time estimation ability.

## Competing interests

The authors declare that they have no competing interests.

## Authors’ contributions

SA and KM conceived the study. SA performed the experiments and the statistical analysis and participated in drafting the manuscript. SH, HS, KK, ME, TS, YA, SK, and AH performed the experiments. SA, HS, KK, and TS participated in study design and its coordination. KM helped with formulating the study hypotheses and drafting of the manuscript. All authors read and approved the final manuscript.
